# Gestational Weight Gain and Body Mass Index in Children: Results from Three German Cohort Studies

**DOI:** 10.1371/journal.pone.0033205

**Published:** 2012-03-22

**Authors:** Andreas Beyerlein, Ina Nehring, Peter Rzehak, Joachim Heinrich, Manfred J. Müller, Sandra Plachta-Danielzik, Martin Wabitsch, Melanie Weck, Hermann Brenner, Dietrich Rothenbacher, Rüdiger von Kries

**Affiliations:** 1 Institute of Social Paediatrics and Adolescent Medicine, Ludwig-Maximilians University of Munich, Munich, Germany; 2 Institute of Epidemiology I, Helmholtz Zentrum München, German Research Center for Environmental Health, Neuherberg, Germany; 3 Institute of Human Nutrition and Food Science, Christian-Albrechts University, Kiel, Germany; 4 University Hospital Ulm, Ulm, Germany; 5 German Cancer Research Center, Heidelberg, Germany; 6 Institute of Epidemiology and Medical Biometry, University of Ulm, Ulm, Germany; Indiana University, United States of America

## Abstract

**Introduction:**

Previous studies suggested potential priming effects of gestational weight gain (GWG) on offspring’s body composition in later life. However, consistency of these effects in normal weight, overweight and obese mothers is less clear.

**Methods:**

We combined the individual data of three German cohorts and assessed associations of total and excessive GWG (as defined by criteria of the Institute of Medicine) with offspring’s mean body mass index (BMI) standard deviation scores (SDS) and overweight at the age of 5–6 years (total: n = 6,254). Quantile regression was used to examine potentially different effects on different parts of the BMI SDS distribution. All models were adjusted for birth weight, maternal age and maternal smoking during pregnancy and stratified by maternal pre-pregnancy weight status.

**Results:**

In adjusted models, positive associations of total and excessive GWG with mean BMI SDS and overweight were observed only in children of non- overweight mothers. For example, excessive GWG was associated with a mean increase of 0.08 (95% CI: 0.01, 0.15) units of BMI SDS (0.13 (0.02, 0.24) kg/m^2^ of ‘real’ BMI) in children of normal-weight mothers. The effects of total and excessive GWG on BMI SDS increased for higher- BMI children of normal-weight mothers.

**Discussion:**

Increased GWG is likely to be associated with overweight in offspring of non-overweight mothers.

## Introduction

Excessive gestational weight gain (GWG) is known to be associated with unfavourable pregnancy outcomes such as pre-eclampsia, macrosomia and emergency caesarean delivery (1–5), whereas insufficient GWG appears to be a risk factor for a number of adverse pregnancy outcomes such as small for gestational age births [1], [2]. Both short- and long- term maternal post-partum weight retention are associated with high GWG [3], [4], [5], [6], [7]. Furthermore, potential priming effects of high GWG on offspring’s overweight in later life have also been discussed recently. Most studies (performed in Europe and USA) agree that there is a positive association between GWG and an increased risk for offspring’s overweight [8], [9], [10], [11], [12], [13], [14]. However, only four of these studies stratified for maternal body mass index (BMI) before pregnancy. While significant associations between high GWG and offspring’s obesity were observed in normal-weight mothers, similar trends did not consistently reach significance for offspring of mothers in other weight categories [10], [12], [13], [14].

The US Institute of Medicine (IOM) established GWG recommendations for women by different BMI classes in 1990 [15], which were slightly revised in 2009 [16]. With respect to the long term effects on childhood obesity adherence might be of particular importance for children of overweight and obese mothers, whose risk for later childhood obesity is increased irrespective of GWG [17]. Previous studies suggested that most environmental risk factors for childhood overweight might not only account for a shift of the total BMI distribution, but have a stronger effect in overweight children compared to normal-weight peers (18–20). To our knowledge, however, no study assessed whether high GWG is associated with BMI in a similar way.

We combined individual data of three German cohort datasets in order to assess whether there is a consistent effect of GWG on offspring’s overweight at 5–6 years across maternal weight groups and whether the effect of GWG on childhood overweight is consistent by the child’s BMI percentile.

## Methods

The setting of the compulsory school entry health examination (SEH) was used in the city of Kiel, Northern Germany, to recruit children for the Kiel Obesity Prevention Study (KOPS) between 1996 and 2001. Children and parents were directly contacted at the SEH. There were no eligibility criteria except willingness to participate. Due to limited personnel power of the KOPS team, only a part of SEHs could be accompanied and thus used for study recruitment (54.6% of the 12,254 children who participated in the SEH in Kiel between 1996 and 2001 were contacted). Targeted SEHs were chosen randomly in different districts in Kiel without preferential selection of schools. Of those who were contacted, 74.7% agreed to participate in the study resulting in the collection of data on n = 4,997 children. The total age range was 52–93 months, with 99% of the observations in the age of 63–88 months. This population was shown to be representative for all children in Kiel entering the SEH in these years [18]. In addition, mothers self-reported their own height and weight and filled out a questionnaire about sociodemographic variables and lifestyle habits such as smoking during pregnancy. As in a previous study [12], information on GWG and offspring’s birth weight was retrieved from maternal interview. More details on KOPS have been described elsewhere [19], [20].

The LISA study is an ongoing population-based birth cohort study of infants, designed to assess ‘Influences of Lifestyle related Factors on the Immune System and the Development of Allergies in Childhood’. Between November 1997 and January 1999, n = 3,097 healthy full-term newborns were recruited from 14 obstetrical clinics in Munich, Leipzig, Wesel, and Bad Honnef. Details on study design are published elsewhere [21], [22]. Information on GWG and the newborn’s weight and length at birth was extracted from the maternity booklet which is issued to every woman during her first pregnancy in Germany. Children were followed up at the age of 5 years where their height and weight were either measured or self-reported by their parents. We *a priori* excluded the data of three children younger than 50 months, leaving an age range of 52–93 months, with 99% of the children in the age of 60–88 months.

The Ulm Birth Cohort Study (Ulmer Säuglingsstudie) is an ongoing population-based birth cohort study which was initially designed to assess infection with *Helicobacter pylori*. Details of the study have been described elsewhere [23], [24], [25]. In brief, all women who delivered at the Department of Gynecology and Obstetrics at the University of Ulm between November 2000 and November 2001 were asked to participate in the study (exclusion criteria: gestational age<32 weeks, birth weight<2000 g, transfer of the newborn to inpatient pediatric care immediately after delivery), resulting in a total of 1,066 mothers and their newborns participating. Information on GWG and the newborn’s weight and length at birth was extracted from the maternity booklet. Information on sociodemographic factors and lifestyle habits was provided by the mothers at baseline via questionnaire. At follow-up both the parents and the paediatrician were asked to fill out a questionnaire, amongst others reporting weight and height of the child.

We calculated children’s and maternal BMI based on respective height and weight records. Children’s BMI was further transformed to sex- and age-specific standard deviation scores (SDS; i. e. z-scores) of the World Health Organisation (WHO) [26]. In accordance with European recommendations [27], overweight and obesity were defined as a BMI above the 90^th^ and 97^th^ reference percentile, respectively. Maternal BMI and GWG were categorised with respect to the current IOM criteria [16]: According to these, underweight women (BMI<18.5 kg/m^2^) should gain 12.5–18.0 kg during their pregnancy, normal-weight women (BMI: 18.5–24.9 kg/m^2^) 11.5–16.0 kg, overweight women (BMI: 25.0–29.9 kg/m^2^) 7.0–11.5 kg and obese women (BMI≥30.0 kg/m^2^) 5.0–9.0 kg. The IOM refers to GWG below/above these ranges as ‘inadequate’ and ‘excessive’, respectively.

The total sample size of all three studies combined was therefore n = 9,105. This number was reduced by exclusion of all children with missing BMI SDS values (n = 1,425), missing information on GWG (additional n = 1,265), maternal BMI (additional n = 87) and birth weight, maternal age and maternal smoking in pregnancy (additional n = 74), resulting in a final sample size of n = 6,254 (KOPS: n = 3,678, LISA: n = 1,937, Ulm Birth Cohort Study: n = 639).

We assessed mean effects of total, inadequate and excessive GWG (with average GWG as the reference) on children’s BMI in linear regression models. Crude and adjusted odds ratios (OR) for offspring’s overweight and obesity were calculated in logistic regression models. Further, potentially different effects on different parts of the BMI distribution were examined in quantile regression models. Quantile regression is a statistical approach of modelling different sample percentiles (‘quantiles’) of an outcome variable with respect to covariates. This approach has been described in more detail elsewhere [28], [29]. Briefly, the interpretation of quantile regression is similar to that of linear regression, but while linear regression models the mean of the outcome distribution, quantile regression models selected quantiles, e.g. the 0.90 quantile (90^th^ percentile). In this study, we analysed the 0.10, 0.30, 0.50 (median) 0.70, 0.90 and 0.97 quantiles. All models were stratified by maternal BMI category and further adjusted for the potential confounders: birth weight, maternal age and maternal smoking during pregnancy, which were recorded in all three studies. In sensitivity analyses, additional adjustment for maternal BMI (as a continuous variable) was performed. In an additional sensitivity analysis, we adjusted for the individual studies in order to detect potential bias by the differing study populations.

All calculations were carried out with the statistical software R 2.12.1 (http://cran.r-project.org), using the *quantreg* package.

## Results

The mean age of the children analysed at the time of examination was 5.9 years with a standard deviation (SD) of 0.6 years (table 1). Children’s BMI had a mean of 15.6 kg/m^2^ and an SD of 1.8 kg/m^2^. The transformation of their BMI values to SDS worked apparently well, resulting in a mean SDS of 0.1 with an SD of 1.1 (ideally, a mean of 0.0 and an SD of 1.0 would be expected after SDS transformation). These observations imply that relative to the WHO standards (26), the study children had slightly higher mean BMI (about 0.16 kg/m^2^ above the WHO standard) and a 10% larger standard deviation in their BMIs. Therefore, one unit of BMI SDS corresponded with about 1.6 kg/m^2^ of ‘real’ BMI. Mean maternal BMI at the beginning of pregnancy was 22.6 kg/m^2^, with 18.5% of the women being overweight or obese at this time. Mean GWG in the data analysed was 14.3 kg, and 37.0% of the mothers analysed had excessive GWG according to the IOM criteria. In the observations excluded, slightly higher mean values (p<0.01) were observed with respect to offspring’s age (6.3 years) and BMI SDS (0.3) as well as for maternal BMI (23.0 kg/m^2^) and GWG (14.8 kg).

**Table 1:** Description of the study population (total: n = 6,254).

	Mean (SD)			
	KOPS (n = 3,678)	LISA (n = 1,937)	Ulm Birth Cohort (n = 639)	Total
Children’s age [years]	6.2 (0.4)	5.2 (0.2)	6.0 (0.1)	5.9 (0.6)
Children’s BMI SDS	0.2 (1.1)	-0.1 (0.9)	-0.2 (1.0)	0.1 (1.1)
Birth weight [kg]	3.4 (0.6)	3.5 (0.4)	3.4 (0.5)	3.4 (0.5)
Maternal BMI [kg/m^2^]	22.5 (3.8)	22.6 (3.8)	23.0 (3.8)	22.6 (3.8)
Maternal age at delivery [years]	27.9 (4.8)	29.0 (4.4)	31.9 (4.5)	28.6 (4.8)
Gestational weight gain [kg]	14.2 (6.8)	14.4 (4.8)	14.7 (5.1)	14.3 (6.1)
	**n (%)**			
Male children	1,857 (50.5%)	997 (51.5%)	322 (50.4%)	3,176 (50.8%)
Overweight (including obese) children	582 (15.8%)	143 (7.4%)	38 (5.9%)	763 (12.2%)
Obese children	308 (8.4%)	45 (2.3%)	18 (2.8%)	371 (5.9%)
Underweight mothers (BMI < 18.5 kg/m^2^)	294 (8.0%)	112 (5.8%)	27 (4.2%)	433 (6.9%)
Normal-weight mothers (BMI: 18.5–24.9 kg/m^2^)	2,716 (73.8%)	1483 (76.6%)	467 (73.1%)	4,666 (74.6%)
Overweight mothers (BMI: 25.0–29.9 kg/m^2^)	485 (13.2%)	249 (12.9%)	108 (16.9%)	842 (13.5%)
Obese mothers (BMI ≥ 30.0 kg/m^2^)	183 (5.0%)	93 (4.8%)	37 (5.8%)	313 (5.0%)
Maternal smoking in pregnancy	1,069 (29.1%)	255 (13.2%)	53 (8.3%)	1,377 (22.0%)
Inadequate GWG	1,179 (32.1%)	405 (20.9%)	131 (20.5%)	1,715 (27.4%)
Excessive GWG	1,338 (36.4%)	699 (36.1%)	275 (43.0%)	2,312 (37.0%)

In linear models without adjustment, total GWG was associated with a slight increase (0.02 or 0.03 SDS units (corresponding with 0.03 to 0.05 kg/m^2^ of BMI) per kg GWG) in mean BMI SDS in children of underweight, normal-weight and obese mothers (table 2). These associations attenuated after adjustment for potential confounders, but were still significant (p<0.05) in offspring of under- and normal-weight mothers. Excessive GWG was associated with a mean increase of 0.08 (95% CI: 0.01, 0.15) units of BMI SDS (0.13 (0.02, 0.24) kg/m^2^), in adjusted models for children of normal-weight mothers. In all subgroups, positive associations of excessive GWG and BMI SDS were found, but these were significant in normal-weight mothers only. With respect to inadequate GWG, no significant associations with offspring’s mean BMI SDS were found in adjusted models.

**Table 2:** Linear regression estimates [95% confidence intervals] for the association of total, excessive and inadequate gestational weight gain (GWG) with offspring’s BMI SDS at 5–6 years.

		Underweight mothers (n = 433)	Normal-weight mothers (n = 4,666)	Overweight mothers (n = 842)	Obese mothers (n = 313)
GWG [kg]	Unadjusted	**0.03** [0.01, 0.04]	**0.02** [0.02, 0.03]	0.01 [0.00, 0.02]	**0.03** [0.01, 0.05]
	Adjusted	**0.02** [0.00, 0.03]	**0.01** [0.01, 0.02]	0.01 [-0.01, 0.02]	0.02 [0.00, 0.04]
Excessive GWG	Unadjusted	0.24 [-0.02, 0.49]	**0.17** [0.10, 0.24]	0.14 [-0.04, 0.32]	**0.42** [0.02, 0.83]
	Adjusted	0.16 [-0.09, 0.41]	**0.08** [0.01, 0.15]	0.09 [-0.08, 0.27]	0.34 [-0.07, 0.74]
Inadequate GWG	Unadjusted	-0.19 [-0.40, 0.02]	-0.03 [-0.11, 0.04]	0.08 [-0.20, 0.37]	0.05 [-0.52, 0.62]
	Adjusted	-0.13 [-0.34, 0.07]	0.00 [-0.07, 0.07]	0.05 [-0.24, 0.33]	0.06 [-0.51, 0.62]

Models were calculated both unadjusted and adjusted for birth weight, maternal age and maternal smoking during pregnancy, and were additionally stratified by maternal pre-pregnancy BMI. Estimates written in bold font denote significant associations (p<0.05).

Similar conclusions could be drawn from analyses assessing ORs for offspring’s overweight (including obesity): While crude analyses suggested potential effects of total and excessive GWG on overweight of offspring of underweight, normal-weight and obese mothers, these associations attenuated after adjustment and were only significant in normal-weight mothers (table 3). Again, no associations with inadequate GWG were found. Analyses with offspring’s obesity as an outcome resulted in similar findings (data not shown).

**Figure 1:**
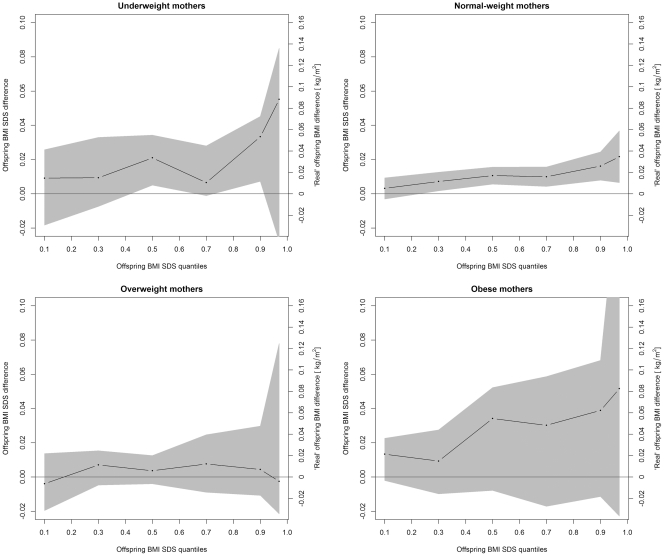
Quantile regression estimates and 95% confidence bounds (grey areas) for the associations of total gestational weight gain (GWG) with selected quantiles of offspring’s BMI SDS at 5–6 years (adjusted models). The dots represent quantile regression estimates at the 0.1, 0.3, 0.5, 0.7, 0.9 and 0.97 quantiles of BMI SDS and are connected by dashes to visualise trends by BMI SDS quantiles. A horizontal line depicts the y = 0 reference.

Quantile regression analyses suggested that the effects of total GWG on BMI SDS increased by BMI SDS percentiles in children of normal-weight mothers, but no significant patterns were found in children of mothers from other BMI groups (figure 1). An increase of 1 kg in total GWG was associated with a shift of 0.00 (0.00, 0.01) units of BMI-SDS at the 0.10 quantile (0.00 (0.00, 0.02) kg/m^2^), of 0.01 (0.00, 0.02) units (0.02 (0.00, 0.03) kg/m^2^) at the median, of 0.02 (0.01, 0.03) units (0.03 (0.02, 0.05) kg/m^2^) at the 0.90 quantile and of 0.02 (0.00, 0.04) units (0.03 (0.00, 0.06) kg/m^2^) at the 0.97 quantile of the outcome variable. Excessive GWG was associated with a shift of −0.03 (−0.08, 0.02) units of BMI-SDS at the 0.10 quantile (−0.05 (−0.10, 0.03) kg/m^2^), of 0.09 (0.01, 0.18) units (0.14 (0.02, 0.29) kg/m^2^) at the median and of 0.16 (0.01, 0.31) units (0.26 (0.02, 0.50) kg/m^2^) at the 0.90 quantile, while no significant association was found with respect to the 0.97 quantile (0.14 (−0.07, 0.35) SDS units; 0.22 (−0.11, 0.56) kg/m^2^). No such patterns were found in underweight, overweight and obese mothers (data not shown).

**Table 3:** Odds ratios [95% confidence intervals] for the associations of total, excessive and inadequate gestational weight gain (GWG) with offspring’s overweight (including obesity) at 5–6 years.

		Underweight mothers (n = 433)	Normal-weight mothers (n = 4,666)	Overweight mothers (n = 842)	Obese mothers (n = 313)
GWG [kg]	Unadjusted	**1.08** [1.02, 1.14]	**1.04** [1.03, 1.06]	1.02 [0.99, 1.04]	**1.04** [1.00, 1.07]
	Adjusted	**1.06** [1.00, 1.12]	**1.02** [1.01, 1.04]	1.01 [0.99, 1.03]	1.03 [0.99, 1.07]
Excessive GWG	Unadjusted	**3.02** [1.15, 8.50]	**1.57** [1.26, 1.97]	1.21 [0.81, 1.82]	**1.97** [1.04, 3.92]
	Adjusted	2.63 [0.98, 7.54]	**1.28** [1.02, 1.61]	1.11 [0.74, 1.68]	1.80 [0.94, 3.64]
Inadequate GWG	Unadjusted	0.86 [0.29, 2.56]	1.11 [0.87, 1.41]	0.95 [0.47, 1.82]	1.06 [0.40, 2.71]
	Adjusted	0.93 [0.31, 2.80]	1.17 [0.91, 1.50]	0.89 [0.44, 1.71]	1.07 [0.40, 2.79]

Models were calculated both unadjusted and adjusted for birth weight, maternal age and maternal smoking during pregnancy, and were additionally stratified by maternal pre-pregnancy BMI. Estimates written in bold font denote significant associations (p<0.05).

Additional adjustment for maternal BMI (continuous variable) did not considerably affect the adjusted estimates in any model; neither did adjustment for the individual studies (data not shown).

## Discussion

Excessive GWG was associated with a 28% increased risk for childhood overweight in normal weight mothers. Although the point estimates varied by maternal BMI strata, the 95% CIs overlapped widely suggesting that there is no substantial difference regarding this effect by maternal BMI stratum.

In children of normal-weight mothers, a 1 kg change in GWG shifted the mean ‘real’ BMI by 0.02 kg/m^2^, while the 90^th^ percentile was shifted by 0.03 kg/m^2^. Excessive GWG, which has been observed with increasing frequency in recent years [30], [31], accounted for an average increase in BMI of 0.13 kg/m^2^. Although these differences are small, they may well be relevant on a population level, as the associations of children’s BMI with smoking in pregnancy and formula-feeding have been found to be of similar size [29].

Excessive GWG appeared to be slightly more pronounced at higher BMI SDS percentiles, as demonstrated by quantile regression. The respective linear regression coefficients, indicating shifts in mean BMI, were also significant. The latter result is in accordance with the quantile regression analyses: The effect on the mean BMI is expected to reflect an ‘average’ of the different associations at lower and higher percentiles.

These findings confirm previous studies which had also suggested weak potential effects of GWG on offspring’s body composition [8], [9], [10], [11], [12], [13], [14]. In those studies with stratification for maternal pre-pregnancy BMI, meaningful associations appeared likely in offspring of non-overweight mothers, but no conclusive findings could be drawn with respect to children of overweight and obese women [10], [12], [13], [14]. However, the effect estimates suggested also positive associations in the latter subgroups both in the mentioned studies and this one, indicating insufficient statistical power for the respective subgroup analyses in the individual studies.

While excessive GWG was found to be associated with higher BMI/overweight in the offspring of normal-weight mothers – and possibly in all subgroups – no potential effects were detected for inadequate GWG. This is in accordance with previous studies [10], [12] and suggests a potential threshold effect for the association of GWG and offspring’s overweight. Whether the definition of excessive GWG as defined by the IOM corresponds with the “true” threshold, is not clear, however.

Interestingly, our analyses suggested different effects of GWG on different parts of the BMI distribution in children of normal-weight mothers. It appears therefore possible that although intervention programs aiming to reduce high GWG would induce lower BMI values in the entire offspring population, (later) heavy-weight children would potentially have the greatest benefit [29].

Although there are determinants of GWG which cannot be influenced such as maternal age, height and parity [16], some might be amenable to interventions. Two recent meta-analyses showed that intervention programs to increase physical activity during pregnancy or a combination of physical activity and diet counselling might reduce GWG slightly [32], [33].

### Strengths and difficulties

Aggregating three cohorts allowed us to analyze more than 6,000 mother child dyads which strengthens the findings of our study. Using different study populations did not affect the results as shown in a sensitivity analysis. Most of the anthropometric data were abstracted from records (maternity pass) or measured providing valid information. In some cases maternal pre-pregnancy BMI or children’s height and weight were self-reported which may be a potential source of bias. Former analyses, however, showed that self- reported pre-pregnancy weight is significantly related to measured weights [34]. If women tended to slightly overestimate their GWG as a recent study suggested [35], our results would probably have been biased towards the null, as only a part of the data used in this study (those from KOPS) was based on self- reported GWG.

The relatively high exclusion rate of about 30% of the entire cohort is not likely to have biased our results: The observations analyzed had only slightly different values with respect to the key variables compared to the observations excluded.

Unfortunately, we were not able to adjust for child’s diet, physical activity or TV viewing time, which represent important risk factors of childhood overweight. However, we do not think that this constitutes a major limitation of our study, since these factors appear unlikely to influence the association between GWG and childhood’s body composition, as they appear a long time after pregnancy. This might be more likely for factors associated with the respective pregnancy such as maternal age, maternal smoking in pregnancy and birth weight, for which we were able to adjust in our analyses.

Conclusion: Interventions limiting GWG might have a particularly strong effect on children who become overweight later in life. Adhering to the IOM recommendations for GWG reduces the BMI of the offspring of normal-weight mothers. With respect to offspring of underweight, overweight and obese mothers, a meta-analysis of studies providing effect estimates stratified by maternal weight category seems to be needed.
